# Mineralization of Biomaterials for Bone Tissue Engineering

**DOI:** 10.3390/bioengineering7040132

**Published:** 2020-10-20

**Authors:** Xinchen Wu, Kierra Walsh, Brianna L. Hoff, Gulden Camci-Unal

**Affiliations:** 1 Biomedical Engineering and Biotechnology Program, University of Massachusetts Lowell, Lowell, MA 01854, USA; Xinchen_Wu@student.uml.edu; 2 Department of Chemical Engineering, University of Massachusetts Lowell, Lowell, MA 01854, USA; Kierra_Walsh@student.uml.edu (K.W.); Brianna_Hoff@student.uml.edu (B.L.H.); 3 Department of Biological Sciences, University of Massachusetts Lowell, Lowell, MA 01854, USA; 4 Department of Chemistry, University of Massachusetts Lowell, Lowell, MA 01854, USA; 5 Department of Surgery, University of Massachusetts Medical School, Worcester, MA 01655, USA

**Keywords:** bone, tissue engineering, mineralization, biomaterial, scaffold

## Abstract

Mineralized biomaterials have been demonstrated to enhance bone regeneration compared to their non-mineralized analogs. As non-mineralized scaffolds do not perform as well as mineralized scaffolds in terms of their mechanical and surface properties, osteoconductivity and osteoinductivity, mineralization strategies are promising methods in the development of functional biomimetic bone scaffolds. In particular, the mineralization of three-dimensional (3D) scaffolds has become a promising approach for guided bone regeneration. In this paper, we review the major approaches used for mineralizing tissue engineering constructs. The resulting scaffolds provide minerals chemically similar to the inorganic component of natural bone, carbonated apatite, Ca_5_(PO_4_,CO_3_)_3_(OH). In addition, we discuss the characterization techniques that are used to characterize the mineralized scaffolds, such as the degree of mineralization, surface characteristics, mechanical properties of the scaffolds, and the chemical composition of the deposited minerals. In vitro cell culture studies show that the mineralized scaffolds are highly osteoinductive. We also summarize, based on literature examples, the applications of 3D mineralized constructs, as well as the rationale behind their use. The mineralized scaffolds have improved bone regeneration in animal models due to the enhanced mechanical properties and cell recruitment capability making them a preferable option for bone tissue engineering over non-mineralized scaffolds.

## 1. Introduction

### 1.1. Engineering Bone Scaffolds

The major functions of bone include assisting with movement, providing skeletal support, protection of critical organs, production of blood cells, mineral storage and homeostasis, and blood pH regulation. These important functions make bone repair and regeneration critical to restoring patient functionality following bone injury or damage [1]. 

During the natural process of bone repair, the first thing to form is a hematoma at the site of fracture or bone loss. This hematoma serves as the source of various growth factors (interleukin-6 (IL-6), insulin-like growth factor (IGF), transforming growth factor β (TGF-β), fibroblast growth factor (FGF), and vascular endothelial growth factor (VEGF)) for bone regeneration [2]. These growth factors promote the recruitment of mesenchymal stem cells that are committed to the osteoblast lineage, a process mediated by canonical Wnt/β-catenin pathway [3,4]. The site then undergoes both intramembranous and endochondral ossification to form a mineralized callus as a result of the osteogenic cell activities [5]. The mature bone tissue is ultimately regenerated due to the growth and remodeling of this callus [6]. During the beginning of the bone repair process, the recruited mesenchymal stem cells, osteoprogenitor cells and osteoblasts, need to attach to an osteoconductive scaffold for cell activities to proceed [7]. The absence of such scaffold, whether it be natural or artificial, will hinder bone regeneration and fibrous tissue may fill the defect prior to bone tissue [8]. In circumstances where a scaffold composed of the natural matrix, such as when there are remaining bone fragments and periosteum, is not available a tissue engineered scaffold is indispensable for bone regeneration.

Natural bone consists of an extracellular matrix comprised primarily of collagen fibers, hydroxyapatite (HA), interstitial fluid, and cells [9,10]. Approximately 10% to 30% of bone is comprised of a porous, hard, outer layer (i.e., cortical bone), and the remaining 70% to 90% is comprised of a porous, inner layer (i.e., cancellous bone) [11]. Closely matching these properties of natural bone with engineered implants is a great challenge when repairing bone defects, as the closer the resemblance, the greater the chance of the scaffold being accepted by the body and promoting new tissue growth. As the properties of the outer and inner layers of bone are dramatically different, an ideal bone scaffold needs to meet varied requirements of mechanical strength and physical structure. The mechanical properties that close to natural bone will be beneficial for bone scaffolds. [12]. Young’s modulus varies from 18.6 to 20.7 GPa for cortical bone and 10.4 to 14.8 GPa for cancellous bone [13]. The scaffold should also be osteoinductive and osteoconductive so that the osteoblasts can adhere and form new bone [14]. A biomimetic mineral matrix, which increases osteoinductivity and osteoconductivity, is thus indispensable for proliferation and differentiation of osteoblasts. The scaffold should also support angiogenesis, as vessels are needed to transport nutrients and oxygen to, and waste products away from the developing, and eventually matured, bone [15,16]. Thus, a multi-scale, porous scaffold, with a pore size of at least 100 µm, should be considered as that conveniently allows for blood vessel development and matches natural bone structure [17]. An ideal scaffold should also be biodegradable and biocompatible in vivo [12], provide micro-architectures that are similar to natural bone [18], and should degrade at a rate matching that of the bone regeneration in the host, in order to provide sufficient space for continued tissue growth [19].

### 1.2. Conventional Scaffolds for Bone Tissue Engineering

Traditionally, bone grafts such as autografts and allografts, have been used to treat diseased, degenerated, or defective bone. Although the utilization of bone grafts has had limited success in clinical practice, these costly procedures have complications, including risk of infection, graft rejection, and weak osteoinductivity. Therefore, there is a clinical demand for safe, cost-effective, and reliable alternatives to the current bone grafting materials and techniques [20]. 

**Figure 1:**
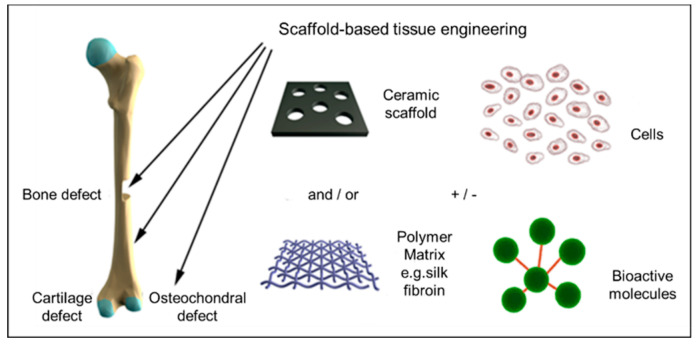
Schematic diagram of skeletal tissue regeneration via scaffold-based tissue engineering strategies. The tissue engineering triad can be concluded as using a combination of cells, growth factors, and scaffolds. Adapted with permission from [22].

To provide a safer and more effective alternative, the development of bone tissue scaffolds has been at the forefront of tissue engineering research for the past few decades [21]. The currently available methods for creating scaffolds use natural or synthetic materials, or a combination of the two [22,23] (Figure 1). Some of the commonly used natural materials include starch-based polymers [24], chitosan [25], collagen [26], alginate [27], and calcium phosphate (CaP) of organic origin [28,29,30]. Synthetic materials used in scaffolds include polyethylene glycol (PEG), polycaprolactone (PCL), poly(lactic-co-glycolic acid) (PLGA), urethanes, calcium phosphates of inorganic origin [31,32], and calcium carbonate [33,34]. The primary techniques used to fabricate conventional scaffolds from these materials are 3D printing, solvent casting, freeze-drying, gas foaming, and electrospinning [35,36]. 

### 1.3. Limitations of Currently Available Scaffolds

One of the major challenges of generating bone scaffolds is developing a biomaterial that has characteristics and functions similar to natural bone. The factors associated with scaffold functionality, such as porosity, surface and mechanical properties, biocompatibility, and biodegradability are also very important for developing artificial bone scaffolds. Conventional scaffolds often are limited in these factors. For example, a biocompatible and pliable polymer-based scaffold may not be able to induce osteogenic differentiation, and the ceramic scaffolds may be too brittle for some applications [27,30]. Due to these limiting factors, many of the traditional methods of fabrication and resulting scaffolds may not meet expectations. 

Several factors contribute to the poor long-term performance of currently available bone scaffolds. First, the incongruity of the mechanical properties of the scaffolds and the bones they are connecting to can cause stress, thereby hampering bone resorption in the long-term [37]. Second, a lack of appropriate interactions, depending on porosity and surface properties such as roughness, between the scaffolds and tissue microenvironments results in a failure to facilitate bi-directional cellular communication, and thus, tissue growth. For example, non-mineralized hydrogel-based scaffolds are typically unable to strongly integrate into the host bone. Finally, the poor degradability of current scaffold options can be an obstacle to continued tissue growth and regeneration as the scaffold continues to take up space preventing new bone from forming in the scaffolds place [38]. There are scaffolds that are fabricated with materials, for example bioactive glass, ceramics, or decellularized bone, that can overcome some of these limitations [39]. However, none of these single component materials are able to address all of the current limitations of the available scaffolds such as the lack of osteoinductivity and/or adequate mechanical properties, or their failure to mimic the native bone. For example, these scaffolds made of mineral-based materials are not tunable and are brittle. However, this limitation can be compensated for by combination with other polymetric materials. In this study, the approaches that improve upon current, non-mineralized scaffolds via mineralization are summarized.

### 1.4. Mineralized Scaffolds

Researchers have developed techniques for growing or incorporating hydroxyapatite into biomaterial scaffolds to enhance bone regeneration [40]. The scaffolds fabricated with this biomimetic approach are referred to as “mineralized scaffolds”. For the sake of this paper we will not be considering scaffolds already made of mineral materials, for example decellularized scaffolds, to be “mineralized scaffolds”. The mineralization process facilitates the deposition of minerals in the presence of organic molecules created by osteoblasts. It is a naturally occurring event in biologic systems, although it also can be artificially induced. [41]. For bone tissue engineering, the goal of the artificial mineralization process is the creation of a modified form of hydroxyapatite (carbonated apatite, Ca_5_(PO_4_,CO_3_)_3_(OH)) that is similar to the mineral composition of natural bone [42]. Hydroxyapatite is primarily formed of calcium and phosphate, with a Ca/P ratio close to 1.67, therefore making Ca^2+^ and PO_4_^3−^ the most important ions for mineralization [43]. 

The bioactivity of a specific bone substitute for implantation, as well as its ability to stimulate the natural healing process within the surrounding tissue, can be significantly enhanced by the creation of hydroxyapatite deposition on the scaffold [44,45]. This is because the osteoprogenitor cells, which are a form of mesenchymal stem cells, and osteoblasts are more likely to attach to a mineralized surface, which is rough, than a smooth one with the help of thrombospondins and vitronectin. There will also be an increased secretion of thrombospondins and vitronectin by the already better attached, and therefore more successful cells, furthering their attachment [46,47,48]. The early cell attachment to a mineral matrix, such as a mineralized scaffold, is crucial for bone healing as the differentiated cells are able to keep recruiting cells of the osteoblast lineage by secreting colony stimulating factors (CSFs), TGF-β, and bone morphogenetic proteins (BMPs) [46,49]. The mineral matrix allows osteoblasts to deposit the extracellular collagen that will make up the collagen matrix. Ca^2+^ and PO_4_^3−^ are secreted by vesicles from within the osteoblasts and precipitate in the environment. Thus, new mineralization will be achieved when these hydroxyapatite crystals form on the collagen matrix [50]. Therefore, with the help of the mineralized scaffolds, the cell activity and callus formation are significantly improved for bone regeneration. 

Mineralization can also improve the mechanical properties of a scaffold, which subsequently allows for the promotion of the differentiation of stem cells and/or progenitor cells towards an osteoblastic phenotype [51]. According to Wolff’s law [52], the applied load to a defect site is crucial for new bone development, especially during the maturation of woven to lamellar bone. If a scaffold cannot sustain the minimal load required to achieve this, then certain key stages of cellular development will not be met. However, a stiff material like metal can cause stress shielding that will result in the loosening of the implant and bone resorption causing the implant to fail [53]. Therefore, scaffolds that can provide the proper, and stable, mechanical properties which closely resemble natural bone, are favorable [54]. 

**Figure 2:**
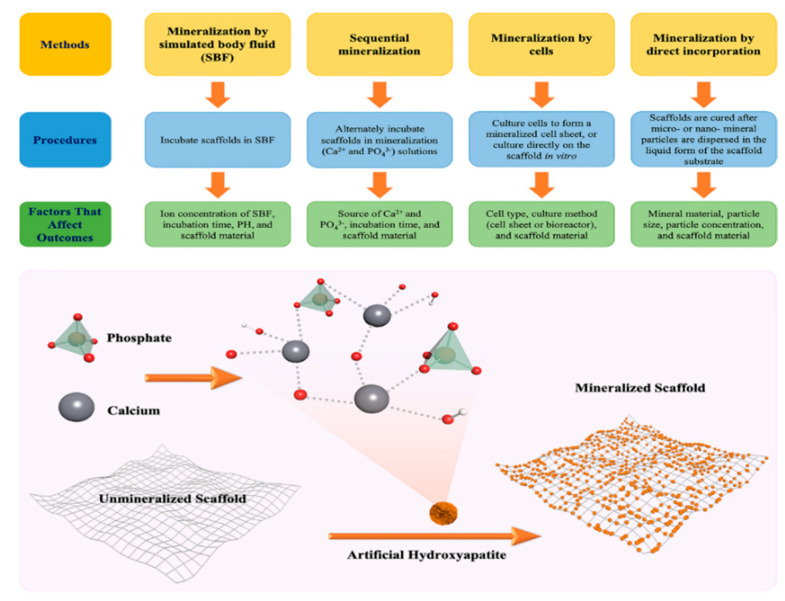
Flow chart for main methods of biomimetic mineralization.

Polymeric scaffolds, especially hydrogels and fibers, have evolved as promising biomaterials for various tissue engineering applications, including bone. These materials are cellularly compatible and hydrated, and have tunable material properties (i.e., size, shape, swelling, degradation, mechanical properties, pore size, porosity). However, on their own, they are typically not suitable for applications in hard tissues such as bone [55]. Naturally derived, synthetic, or composite scaffolds can be modified to simulate bone properties through mineralization. In this review, the fabrication techniques (Figure 2), assessment techniques, and application of scaffolds in bone tissue engineering are summarized. 

## 2. Fabrication of Mineralized Biomaterials

### 2.1. Mineralization by Simulated Body Fluid (SBF)

One approach used for achieving biomimetic mineralization, in order to produce a mineralized scaffold, is to mineralize a polymer construct in simulated body fluid (SBF). SBF is a medium with ion concentrations similar to those of human plasma [56,57]. The ion concentration of an SBF solution affects the amount and growth speed of mineralization of the biomaterial scaffolds. For instance, a mixture of PCL and cellulose acetate (CA), at various compositions, was used to fabricate nanofibrous PCL/CA nonwoven membranes [58]. The membranes were incubated in SBF in order for biomimetic mineralization to occur and the mineral deposition in the resulting mineralized scaffolds was confirmed by X-ray differentiation patterns and Fourier Transform Infrared Spectroscopy. Pre-osteoblasts were then seeded on the mineralized scaffolds, which were shown to be biocompatible by a CCK (Dojindo’s cell counting kit-8) assay which detects the formazan that is produced by the living cells. 

Nanocomposites, such as nanofibers, have also been used to construct bone scaffolds that were then mineralized with SBF. The researchers fabricated poly(lactic acid) (PLA) nanofibers that had been mineralized in SBF [59]. Osteoblast-like (MG63) cells were cultured on these scaffolds and the cell viability was determined using 3-(4,5-dimethylthiazol-2-yl)-2,5-diphenyltetrazolium (MTT) assay, which showed the favorable biocompatibility of the mineralized scaffold. The mineralized scaffolds were then implanted in a rabbit model to treat a femur defect. The in vivo result was consistent with the in vitro study, in that the mineralized scaffold induced significantly more bone formation in the defect compared to the non-mineralized implants. 

Another type of scaffold, which is composed of titanium with a coating of polydopamine (PDA) on its surface, inspired by mussels, facilitated mineralization after incubation in SBF [60]. Calcium phosphate agglomerates were formed on the PDA coated titanium surface and were confirmed to be hydroxyapatite crystals. Another surface coating technique involves the use of negatively charged functional groups, such as hydrogen phosphate (PO_4_H^2−^) or carboxylic acid (COOH^−^), to chemically modify the surfaces of tissue engineered scaffolds. The negatively charged functional groups, and thus modified surface of the scaffold, enhances the minerals’ ability to deposit onto the scaffold. In one study that was conducted, self-assembled monolayers (SAMs) of alkanethiols containing different terminal groups were fabricated into a scaffold. A SAM of alkanethiol that contained PO_4_H^2−^ and COOH^−^ negatively charged groups induced mineralization and HA nucleation after SBF incubation [61]. 

**Figure 3:**
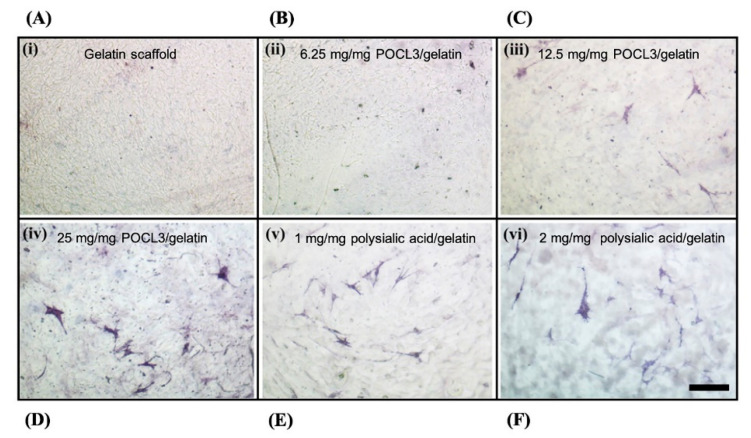
The alkaline phosphatase staining of murine mesenchymal stem cells (C3H10T1/2) on SBF mineralized gelatin scaffolds. (**A**) Gelatin scaffold only. (**B**–**D**) 6.25, 12.5 and 25 mg/mg POCl3 incorporated with gelatin scaffolds, respectively. (**E**,**F**) 1 and 2 mg/mg polysialic acid incorporated with gelatin scaffolds, respectively. The staining showed osteogenic differentiation of stem cells on mineralized scaffolds. Scale bar: 100 μm. Adapted with permission from [11].

Various types of hydrogel-based scaffolds have also been shown to improve with mineralization in SBF. Mineralization in SBF has been achieved in double network (DN) hydrogels, which were fabricated from poly (ethylene glycol) diacrylate (PEGDA) and methacrylated poly(gamma-glutamic acid) (mPGA) [62]. The resulting DN hydrogels exhibited improved mechanical properties (306 kJ/m^3^ in toughness) as well as low cytotoxicity. Hydroxyapatite-like minerals were formed within the DN structure and enhanced the mechanical properties further to make the scaffold suitable for bone regeneration. A gelatin hydrogel was treated by phosphorous oxychloride to form the phosphate ester of gelatin, and the modified hydrogel was then mineralized via SBF [11]. The degree of phosphorylation in the scaffold from the phosphorous oxychloride treatment affected the total mineralization of the scaffold in SBF (Figure 3). 

In another study, PLGA scaffolds were mineralized in SBF and seeded with bone marrow stromal (BMS) cells and adipose-derived adult stromal (ADAS) cells before implantation into cranial defects in mice [63]. Firstly, the mineralized scaffolds greatly enhanced cell attachment and allowed osteoblasts to produce further mineralization on the scaffolds, compared to no further mineralization on the non-mineralized scaffolds. Then, BMS and ADAS were able to induce intramembranous bone formation after 2 weeks of implantation on the mineralized scaffolds. Therefore, the mineralized scaffolds promoted new bone formation, whereas non-mineralized PLGA scaffolds failed to facilitate substantial regeneration. 

Collagen films and some polymeric scaffolds are too fragile to be used as bone scaffolds, and, even if mineralized, the apatite coating formed on their surfaces are susceptible to delamination upon drying [64,65,66,67]. In some of these such cases, the minerals formed by incubation in SBF can disintegrate even under small loads (0.05–1.2 kg) [66]. However, this limitation may be avoided by choosing a scaffold that is able to support mineralization. Mineralization in SBF can take time, but it is possible to speed up the process of mineralization in 5× SBF due to the higher ion concentrations. Concentrated SBF with collagen (1 g/L) has been used to form a collagen-apatite coating on porous poly(L-lactide) (PLLA) films [68]. Researchers have revealed the fact that concentrated SBF can accelerate the mineralization process in vitro. The coating was reported to closely mimic the surface of natural bone. 

### 2.2. Sequential Mineralization via Deposition of Calcium (Ca^2+^) and Phosphate (PO_4_^3−^) 

**Figure 4:**
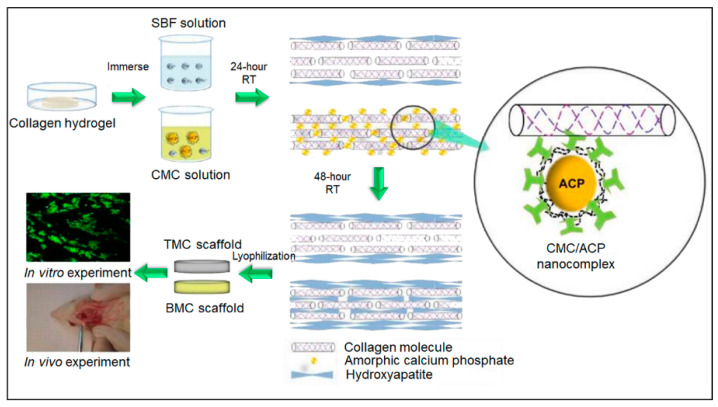
Experimental scheme of the hydroxyapatite formation on the collagen hydrogel scaffold by simulated body fluid (SBF) with the carboxymethyl chitosan (CMC). The biomimetic mineralized collagen (BMC) and traditional mineralized collagen (TMC) were used to repair critical sized cranial defect in a rat model. Adapted with permission from [55].

Another method of mineralization is sequential mineralization, the deposition of minerals through consecutive immersion of the scaffolds in an ionic solution. For the purposes of generating bone scaffolds, these solutions are typically calcium- (Ca^2+^) and phosphate- (PO_4_^3−^) based [55] (Figure 4). Collagen matrices presoaked in a PO_4_^3−^ solution have been subsequently immersed in a Ca^2+^ solution to create crystal polymorphs on the matrices, thus creating mineralized scaffolds. These resulting scaffolds have shown improved osteoconductivity when implanted into a rat model, compared to the bare collagen matrices, as the osteoblasts were able to attach on the mineralized matrix and generate further mineralization. Hutchens et al. (2009) showed the formation of calcium-deficient hydroxyapatite (CdHAp) after sequential incubation of cellulose scaffolds in 100 mM calcium chloride (CaCl_2_) and 60 mM sodium phosphate dibasic (Na_2_HPO_4_) solutions [69]. The elastic modulus and compressive strength were increased by the CdHAp reinforcement and the tensile strength was decreased. The mineralized scaffold also showed a decreased degradation rate *in vitro* which indicated that the CdHAp was chemically bonded to the cellulose fibers.

Through the sequential mineralization of an agarose hydrogel, an enamel-like structure was created, in the absence of cells or enamel proteins [70]. The supply of Ca^2+^ and PO_4_^3−^ ions, from CaCl_2_ and Na_2_HPO_4_ solutions respectively, induced the formation of the enamel-like structure in this particular model. The amorphous precursors were formed by the ions that bound to the organic surface of the hydrogel. Then, the matrix-mediated mesophase transformations induced the oriented crystallization of the precursors and formed the enamel-like mineralization. Sequential incubation of polyacrylic acid (PAA) hydrogels in ammonium phosphate dibasic ((NH_4_)_2_HPO_4_) solution and calcium nitrate (Ca(NO_3_)_2_) solution has also been carried out in order to obtain mineralized biomaterials [71]. A similar method of subsequent treatments in calcium and phosphate solutions was used to prepare mineralized chitosan hydrogels, as they were sequentially soaked first in a CaCl_2_ solution, and then in a Na_2_HPO_4_ solution. The deposition of minerals was then confirmed to be hydroxyapatite in the resulting samples [72]. The biocompatibility of these mineralized scaffolds was confirmed using an MTT assay, which showed the good cell viability for both mineralized and non-mineralized scaffolds, which confirmed the mineralization will not affect the biocompatibility of the substrate scaffold. 

There is also a very similar method to sequential mineralization that generates the mineralization using one solution containing both Ca^2+^ and PO_4_^3−^ ions, rather than using two separate solutions. Gellan gum (GG), which is a negatively charged and naturally derived polymer, has been mineralized through incubation in an aqueous calcium glycerophosphate (CaGP) solution [73]. The study showed that mineralized GG hydrogels are suitable for bone tissue engineering. The negatively charged carboxyl groups in GG causes electrostatic repulsion between the negatively charged groups and the negatively charged alkaline phosphatase (ALP) molecules that are entrapped in the GG hydrogel. This repulsion results in the release of the ALP, which then supports bone formation. The Young’s modulus of the scaffold was increased because of increase in ALP concentration that went along with the mineralization. As the repulsive forces within the GG hydrogel results in the release of the ALP, which then recruits osteogenic cells and guide osteogenic differentiation, and the scaffold has an increased concentration of ALP, the mineralized scaffolds are more osteoinductive. Thus, the mechanically improved and osteoinductive scaffold can further improve the bone formation. ALP was also used in another study to hydrolyze β-glycerophosphate in order to release the inorganic phosphate group, which then further reacted with calcium, from a CaCl_2_ solution, to cause mineralization of collagen films, which served as the scaffold [74]. The calcium phosphate phase was identified and the cementoblasts were cultured on the mineralized scaffolds to show the suitable biocompatibility. 

The deposition of Ca^2+^ and PO_4_^3−^ ions is highly effective in mineralizing biomaterials for bone tissue engineering [67,69,71,72]. However, the source of Ca^2+^ and PO_4_^3−^, and the concentration of each solution, can cause the Ca/P ratio to vary dramatically. Thus, extensive experiments need to be performed to optimize the conditions in order to obtain the favorable Ca/P ratio, close to 1.67, for the mineral created by sequential mineralization.

### 2.3. Mineralization by Cells

Cell sheets of osteogenic lineages can be used to obtain mineralized scaffolds for tissue engineering since certain types of cells naturally deposit minerals in their environment. In thermosensitive Petri dishes, Flores et al. (2008) cultured periodontal ligament (PDL) cells in osteogenesis media. After the cell sheet formed a mineralized layer, the cell sheet was lifted by lowering the temperature of the Petri dish. The mineralized cell sheets, which acted as mineralized scaffolds, were then implanted in rats to repair periodontal fenestration defects. The results showed fibrous tissue formation, PDL cell growth in the mineralized layer of cementum-like tissue, and significant periodontal regeneration [75]. 

**Figure 5:**
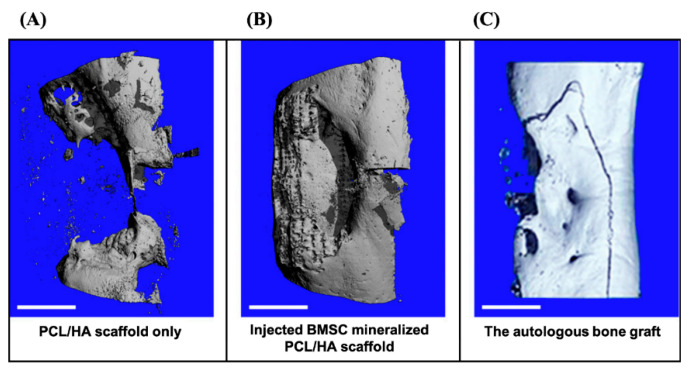
The micro-CT analysis after 3D reconstructions. (**A**) The tibia bone defect was repaired with PCL/HA scaffold only. (**B**) The BMSC cell sheet was injected into the PCL/HA scaffold for in situ mineralization. (**C**) The autologous bone graft (ABG) positive control. Scale bars: 1 cm. Adapted with permission from [78].

Another method for using cells to fabricate mineralized scaffolds involves the culture of bone marrow stromal cells, also known as bone mesenchymal stem cells (BMSCs), on 3D scaffolds in flow perfusion bioreactors. For instance, in one study, BMSCs were isolated from rat bone marrow and then seeded into a PLLA scaffold. The scaffold was then cultured in a flow perfusion bioreactor with a flow rate of 0.6 mL/min. A calcified extracellular matrix was generated on the scaffold and confirmed by an ortho-cresolphtalein complexone method [36,76,77]. The resulting scaffold was able to induce the upregulation of bone sialoprotein, runt related transcription factor 2 (RunX2), and BMP-2 in BMSCs. Another study used BMSC sheets to generate mineralized scaffolds [78] (Figure 5). The formation of minerals within the BMSC sheets by the cells were confirmed by Alizarin red staining after a two-week culture period, prior to the injection of the cell sheets into polycaprolactone/hydroxyapatite (PCL/HA) composite scaffolds, which had been placed in critical-sized tibial defects (3 cm) of sheep. The BMSC sheets were transferred to a syringe using a pipette, injected into the PCL/HA composites to induce mineralization of the scaffold up to a year. After 3, 9, and 12 months, the mineralized scaffolds had significantly improved bone regeneration in comparison to the non-mineralized scaffolds. The Micro-computed tomography (micro-CT) scans showed the complete bridging of newly formed bone tissue, which entirely filled the defect after 12 months. In contrast, the non-mineralized scaffold group still had a void where the defect was. The histology study showed the scaffold was well integrated with the partially regenerated bone close to the native bone, indicating the scaffold was biocompatible. One limitation, however, is that cell sheets are fragile and difficult to handle, which makes it difficult for this mineralization technique to be standardized. Nevertheless, mineralization is necessary for these scaffolds to achieve comprehensive bone regeneration.

### 2.4. Mineralization by Direct Incorporation of Minerals

Minerals can also be directly incorporated into biomaterials to fabricate hybrid scaffolds with reinforced properties. These mineral-reinforced biomaterials have proven to be useful in bone tissue engineering applications [1,79]. For instance, a study incorporated hydroxyapatite into poly(lactide/glycolide) scaffolds in order to obtain osteogenic constructs. The resulting composite scaffolds caused higher levels of cell adhesion and proliferation, as well as inducing higher osteogenic differentiation for the adipose-derived stem cells when compared to the non-mineralized scaffolds [80,81]. 

The generation of composites with mineralized nano- or micromaterials is an effective strategy for improving the mechanical properties of biomaterials and rendering them suitable for bone tissue engineering [82,83]. In a recent study, Wu et al. (2019) reinforced protein-based hydrogels with chicken eggshell microparticles (ESP) for bone tissue engineering [84]. The compressive strength of the hydrogels was significantly improved by the addition of ESP and the scaffolds were demonstrated to be highly osteoinductive. The pre-osteoblasts were encapsulated in the ESP-reinforced hydrogel and pristine hydrogel. After seven days, the ALP activity of the cells was significantly higher in the ESP group than the gel group. The osteocalcin expression was also determined by polymerase chain reaction (PCR) at day 14 as a late osteogenic differentiation maker. The result showed significant higher gene expression in ESP group as well. These studies demonstrate that scaffolds that are reinforced with minerals have improved osteogenic activity.

## 3. Assessment of Mineral Deposition

### 3.1. Quantification of Mineral Deposition

Thermogravimetric analysis (TGA) is a technique where the mass of a sample is measured over time while the sample is heated at a specific rate with controlled airflow [85]. Organic materials usually exhibit mass loss at continuously elevated temperatures, and these mass losses can be characterized by TGA to provide information about the sample’s composition, thermal behavior, and thermal stability. This technique has been used in the characterization of the mineral component of mineralized hydrogels, nanofibers, and other polymeric scaffolds since heating to a certain temperature (dependent on the scaffold material) removes all components of the scaffold other than the minerals, and the remaining mass of minerals is determined. The balance of the instrument determines the sensitivity of TGA, usually 0.1 μg [86], which is sensitive enough to quantify and determine the composition of the mineral deposition on a scaffold. Douglas et al. (2016) used TGA to show that the incorporation of ALP into GG hydrogels induced the formation of an appetite-like material at the sub-micron scale. The GG hydrogels that had ALP and were mineralized experienced better mineralization than the GG hydrogels without ALP that, when mineralized, experienced less mineralization. The mineralized samples with or without ALP were heated up to 800 °C to identify only the mineral weight, as the rest of the scaffold had gasified. The percentage of remaining weight at 800 °C was significantly higher for the GG gels with ALP (50%) than without ALP (25%) after 7 days of mineralization. This showed that there was more mineral growth on the scaffolds with ALP than without, and their study also showed that increasing the concentration of ALP increased the amount of calcium phosphate deposited onto the scaffold. This increase in mineral deposition resulted in an increase in the stiffness of the scaffolds as well [87]. Salama et al. (2017) fabricated a biohybrid material with cellulose grafted soy protein isolate and then mineralized the material in SBF [88]. TGA was performed to assess and confirm the formation of hydroxyapatite after the mineralization process. However, TGA may not always be a feasible characterization technique, specifically if the matrix material of the scaffold has a higher melting range than that of the minerals. 

### 3.2. Characterization of the Chemical Composition

Fourier-transform infrared spectroscopy (FTIR) has been used for the chemical characterization of hydrogels [69,73], fibrous scaffolds, [89,90] and polymeric biomaterials [91] in bone tissue engineering. This technique is utilized to study the chemical functional groups present in the biomaterials [92]. For example, Cao et al. (2013) used FTIR to study artificially created enamel-like structures and demonstrate the presence of phosphate groups, an important component of natural enamel, on the etched enamel surfaces [70]. The phosphate groups showed in the 520 to 660 cm^−1^ and 1037 to 1117 cm^−1^ regions for their sample, which confirmed the presence of HA. Douglas et al. (2014) used FTIR to confirm the formation of CaP in hydrogels in the presence of ALP [73]. FTIR has also been used to provide evidence of CaP formation in enzymatically mineralized collagen gels [55]. Furthermore, Vo et al. (2017) used FTIR to determine the formation of apatite-like minerals in a gelable hydrogel [93]. 

The beam area is much bigger for FTIR compared to dispersive instruments, such as energy-dispersive X-ray spectroscopy (EDS), which means more infrared energy will reach the detector, and therefore the sensitivity is much higher. Due to this high sensitivity, lower concentration samples such as 30 ppm can be analyzed using FTIR. One limitation of regular FTIR is that this method is typically used for qualitative analysis rather than quantitative analysis. However, the attenuated total reflection (ATR)—FTIR is an effective way to perform quantitative analysis for mineralized samples [94].

EDS performs localized chemical analyses by bombarding a solid sample with a focused beam of electrons [95]. EDS is a powerful method for determining the elemental composition of, as well as the relative abundances of the elements in, the material tested. EDS has been used to evaluate the formation of mineral deposits in both hydrogels [9,62,93] and fibrous scaffolds [96,97]. For example, Tomomatsu et al. (2010) used EDS to show that mineral directing gelator (MDG) peptide-based hydrogels induced the formation of hydroxyapatite in the presence of CaCl_2_, β-glycerophosphate, and ALP as previously described [74]. EDS is performed together with scanning electron microscopy (SEM) as researchers need SEM to locate the point of the material that is being analyzed, as well as to generate the electron beam. The EDS resolution is based in the SEM instrument and the result may also be affected by the astigmatic beam [98].

Inductively coupled plasma-optical emission spectrometry (ICP-OES) uses the emission spectrum of a sample to identify, and quantify, the elements that it contains. More specifically, the constituent elements can be identified by their characteristic emission traces, and then quantified by the intensity of those traces [99]. The sensitivity of ICP-OES varies from 0.05 μg/L to 200 μg/L for different elements, and the precision is outstanding (0.3–2% RSD). However, there are limitations when using ICP-OSE for non-metallic elements as they are on the lower end of the sensitivity spectrum. The required liquid form of the sample may also limit the use of this technique in some cases. Using a dissolution method to generate the minerals is essential when considering using ICP-OES. 

In mineralization studies, ICP-OES has been used to confirm the presence of Ca and P, as well as to quantify these minerals [74,88]. For example, Douglas et al. (2017) used ICP-OES to determine the chemical composition of the minerals in the study’s mineralized hydrogels, confirming the presence of Ca, Mg, and P. [100]. In this study, ALP-loaded GG hydrogels were mineralized via incubation in a medium containing Ca glycerolphosphate and/or Mg glycerolphosphate. The effects of the mineralization medias with different ratios of Ca glycerophosphate:Mg glycerophosphate were then compared, also using ICP-OES. 

### 3.3. Characterization of the Crystal Structure

X-ray diffraction (XRD) is a technique used for determining the atomic and molecular structure of a crystallite material [101]. After irradiating samples with X-ray, different X-ray diffractions appear, which represent the different atomic distribution within the sample. XRD has been used to identify and analyze the (002) and (112) typical reflections for crystalline structures of minerals on the surfaces of hydrogels [9,73], polymeric, and fibrous scaffolds [96,97]. The crystalline structure is crucial to examine because it has important implications in rendering the scaffolds more osteoinductive and osteoconductive. Crystalline apatite is more stable environment in that it degrades more slowly than amorphous apatite [102,103]. The slower degradation rate ensures the long-term osteoinductivity and osteoconductivity of the mineralized scaffold and prevents the over-saturation of the environment that can be caused by the quickly dissolving amorphous apatite. The fast degradation of the mineralization can also result in a burst release of the degradation product, which may lead to undesirable outcomes. Moreover, at the beginning of bone regeneration, only amorphous apatite is created by the recruited osteoblastic cells and thus is responsible for the cells’ bonding to an osteoconductive scaffold [7,103]. The cells can hardly form bone tissue with mature crystalline hydroxyapatite without any form of natural or artificial scaffold, such as bone fragments or artificial crystalline hydroxyapatite. 

The presence, and overall orientation, of the crystalline phases of the mineral layers in mineralized scaffolds have previously been evaluated by XRD [9]. XRD was also used to show the presence of HA nanocrystals in hydrogel scaffolds [73], and to differentiate between the crystal structures of fluorapatite (FA) and HA [70]. In another study, Furuichi et al. (2006) fabricated a calcium phosphate-based organic polymer composite, with a hierarchical structure, via the calcification of a poly(acrylic acid) hydrogel. The group then used XRD to demonstrate the formation of layered structures comprised of HA crystals [71]. XRD is known for its sensitivity, resolution, straightforward operational procedure, and speed. XRD is also able to provide quantitative analyses when given the standard reference of a pure material. For some instruments, the powder size of the sample is critical, and this may limit the range of applications if the sample from the scaffold cannot be ground finely enough [104]. The crystal structure that is identified using XRD is crucial in the evaluation of the success of biomimetic mineralization as the HA in natural bone is in crystalline form.

### 3.4. Surface Characterization

**Figure 6:**
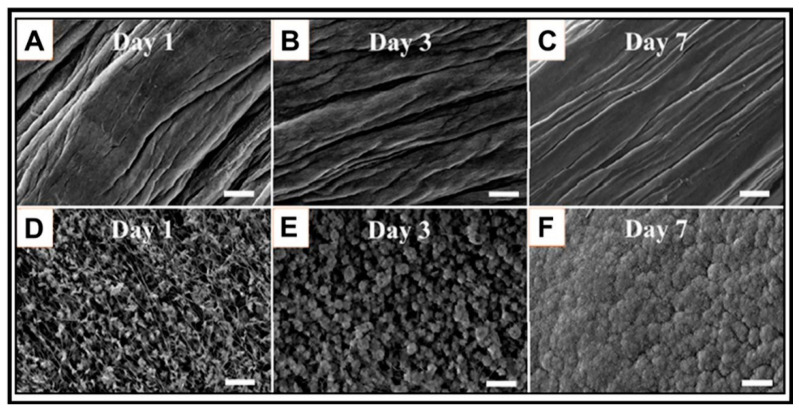
SEM images for the SBF-mineralization of bioglass 45S5-incorporated electrochemically aligned collagen (BG-ELAC) scaffolds. (**D**–**F**) showed increased mineralization crystals compared to the smooth surfaces of (**A**–**C**), respectively. Adapted with permission from [105].

Scanning electron microscopy (SEM) is a conventional electron microscopy approach used for surface structural characterization of materials [105,106] (Figure 6). By scanning the surface with a focused beam of electrons, images of the material’s surface are produced to reveal topographical and compositional information [12]. In another study, the morphology of enamel regeneration based on mineralized agarose hydrogels was characterized by SEM in a time-dependent manner [70]. The continuous growth of prism-like crystals on the enamel was observed every two days. SEM has also been used to determine the layered structure that results from the precipitation of calcium phosphate on cross sections of poly(acrylic acid) hydrogel scaffolds containing ALP [71]. The ALP facilitated the CaP formation in SBF, as shown by the significant increase in the CaP clusters in the SEM images.

Transmission electron microscopy (TEM) is another microscopy technique, using a beam of electrons that transmits through a sample to produce an image. TEM captures details at the atomic level, which provides detailed information, such as about the crystal structure of the minerals forming on the hydrogel, polymeric, or fibrous scaffold that is being imaged [107]. TEM has been used to demonstrate the presence of hydroxyapatite nanoparticles in layered scaffolds. Researchers have fabricated a layered structure using PAA and then mineralized that scaffold using sequential mineralization with Ca^2+^ and PO_4_^3−^ solutions. The formation of hydroxyapatite nanoparticles on the scaffold, without a specific crystallographic orientation, was then confirmed by TEM [71].

Both SEM and TEM provide high resolution images with an optimal spatial resolution around 1 nm and 50 pm, respectively [108]. Because of this, electron microscopy is widely used and considered indispensable for characterizing the surface structure of modified tissue engineering scaffolds. However, the sophisticated sample processing for non-conductive material may limit the use of electron microscopy for some bone scaffolds. Moreover, to expose the cross-section of a tiny structure is difficult and the assistance of other characterization techniques is required [109]. 

### 3.5. High-Resolution 3D Imaging

Micro-CT imaging is a nondestructive imaging method that generates images and measurable parameters of a sample within a 3D space [18,110]. High-resolution micro-CT has been commonly used to image and analyze cancellous and cortical bone morphologies, allowing for their comparison to biomimetic scaffolds [111,112]. This technique can also provide quantitative measurements such as the volume-to-surface ratio of the pores in the scaffolds, which is difficult to obtain using other techniques [112]. Lin et al. (2003) used micro-CT to analyze poly(L-lactide-co-DL-lactide) hydrogel scaffolds and quantified the micro-architectural parameters as a function of the polymer concentration prior to mineralization in order to help predict both mineralization success and compressive mechanical properties post mineralization [113]. With increasing polymer concentrations, the volume fraction decreased consistently due to the micro-architectural changes in the average strut thickness, spacing, and density. The utilization of micro-CT demonstrated that these biodegradable, porous polymer scaffolds with micro-architectural features could facilitate vascular invasion and cellular attachment with biomimetic mechanical properties comparable to those of cancellous bone. Micro-CT provides researchers with a 3D image of the examined sample, and both the volume and density of the scaffold or bone tissue can be quantified. A high-resolution micro-CT can contribute 9 nm resolution. However, the high dosage of X-ray radiation that is associated with micro-CT is not recommended for living animals [114,115].

### 3.6. Mechanical Testing

The mechanical properties, such as compressive strength, tensile strength, and stiffness, of the scaffolds affect the macroscopic mechanical environment that provides the physical stimulation that the bone cells require for regeneration [7]. A successful bone scaffold should be able to provide adequate and stable stiffness that increases the cell migration velocity and then supports callus formation [116,117]. The dynamic mechanical analyzer (DMA) is widely used to determine the mechanical strain and stress response of a sample to applied compressive, and tensile forces [118]. Based on the strain and stress, Young’s modulus (stiffness), compressive strength, and tensile strength, can be determined for scaffolds [119]. For bone scaffolds, the compressive strength and tensile strength represent the maximum force to break the scaffold in compressive and tensile ways, and the Young’s modulus is usually used for indicating the stiffness of materials [120]. 

DMA is a high sensitivity technique for mechanical testing [121]. The ramping force can be set from 0.0001 N to 18 N, and the force resolution is 0.00001 N [122]. DMA is appropriate for analyzing a broad range of materials, in a broad range of temperature (−150 to 600 ℃) [123,124]. For example, Yang et al. (2017) performed mechanical testing of their mineralized double network hydrogel [62]. The compressive testing showed the enhanced stress, strain, and toughness of the mineralized samples. Rauner et al. were able to mineralize double network hydrogels, which were fabricated with triethylene glycol dimethacrylate (TEG) and poly-N,N-dimethyl acrylamide (PDMA), in CaGP solution with the help of ALP [125]. The goal of this study was to increase the stiffness of the hydrogel scaffold to make it more suitable for bone tissue engineering. The physical stimulation such as compressive load is needed for osteogenic lineage cells to differentiate, and the bone scaffold should be able to provide such support [126]. DMA was used to determine the change in Young’s modulus (stiffness) of the scaffolds being mineralized over time. The stiffness was increased after the scaffolds had been mineralized for four days. 

Though the literature has suggested than an increase in the bulk stiffness of a scaffold directly leads to an increase of bone regeneration [127], stress shielding is a concern for metal scaffolds, which have an innately high stiffness. Pobloth et al. designed two mechanical distinct mineralized Ti mesh scaffolds (soft and stiff) based on the mechanical testing technique to show how stiffness affects bone regeneration [128]. The two types of scaffolds were then implanted in a critical sized (4 cm) tibia defect in a sheep model, and the bone regeneration was monitored from four weeks to 24 weeks. The softer scaffold showed less stress shielding effect and achieved earlier and greater bone regeneration than the stiffer scaffold. However, many mineralized scaffolds are not based in a metal scaffold and thus are starting off far softer and require mineralization to reach a level of stiffness that makes them adequately able to support bone regeneration.

## 4. Applications of Mineralized Biomaterials in Bone Tissue Engineering

### 4.1. Mineralized Bone Scaffolds with Improved Osteoconductive and Osteoinductive Activity

Mineralization is an efficient way to improve the mechanical properties and bioactivity of engineered bone scaffolds, thus increasing their osteoconductive and osteoinductive activity. Minerals are stronger than the initial scaffold material, thus enhancing the mechanical properties of the entire scaffold. They also provide better attachment sites for the osteogenic lineage cells to differentiate and proliferate to form new bone tissue. Researchers have mimicked the composition of natural bone through mineralization of scaffolds, prior to their cellularization, in order to fabricate better functioning bone grafts (Table 1). 

Biomimetic scaffolds have mechanical and structural properties that are more supportive to osteogenic lineage cells, their mineral composition supports further natural mineralization by the cells, and these scaffolds have increased acceptance by the body. For example, polyacrylic acid/hydroxyapatite (PAA/HA) composites exhibited mechanical properties that were similar to those of natural bone thus improving the scaffolds’ functionality [71]. 

Various types of bone scaffolds have been fabricated to demonstrate the importance of mineralization [60]. In one study, Ti foil, bio-glass, paper, and poly(methyl methacrylate) (PMMA) were coated with polydopamine and mineralized by SBF, and then seeded with pre-osteoblasts. The mineralized biomimetic surfaces of these scaffolds demonstrated improved cell adhesion. The pre-osteoblasts generated ECM that was attached on the mineralized biomimetic surface and proliferated. The ECM served as new matrix for new cells to attach, as well as allowing for cell-cell communication. The high cell viability showed the non-cytotoxic behavior of the mineralized scaffolds. 

Enzymatically mineralized gellan gum-based hydrogels have also been shown to be suitable for bone regeneration [73]. ALP was loaded into the hydrogels and assisted mineralization in calcium glycerophosphate solution for two and six days. The resulting amount of mineral deposition on the scaffolds was determined using TGA, which showed the ALP improved the mineralization significantly on day six. Human fibroblasts were then cultured on the scaffolds to demonstrate the cytocompatibility using an MTT assay. The in vitro cell culture experiments revealed high cell attachment and proliferation.

Another study showed that mineralized PLGA scaffolds facilitate the osteogenic differentiation of adipose-derived adult stromal (ADAS) cells as the ADAS on the mineralized scaffolds initiated intramembranous bone formation in the critical-size skeletal defects in a mouse model [63]. After 12 weeks, complete bony bridging was formed within the critical sized defect (5 mm) and the new bone consisted of 92–99% implanted stem cells.

Bio-integration of implants into the existing tissue is another crucial parameter that aids in tissue regeneration. For example, a chitosan sponge was fabricated and BMP-7 and pyrophosphatase was encapsulated in the sponge [130]. The pyrophosphatase induced the mineralization of the scaffold. The mineralized scaffold was then injected to fill the 6 mm segmental defect in the femur of a rat model. The minerals and the BMP-7 in the scaffold were shown to serve as a chemoattractant for BMSCs that can guide cell migration. The osteogenic cell recruitment is essential to initiate bone regeneration and mineralization not only attracts cells, but also further improves the cell attachment. Mata et al. (2010) fabricated peptide amphiphile (PA) hydrogels with phosphoserine residues, which can nucleate hydroxyapatite crystals [129]. Arginylglycylaspartic acid (RGD) was also incorporated into the hydrogels to further improve the cell attachment. These mineralized hydrogels were then used to repair critical-sized femoral defects (5 mm) in rats and showed similar, and slightly improved, outcomes compared to allogenic bone grafts. The mineralized RGA-PA hydrogel showed 24.8 mm^3^ of bone volume regenerated while the allograft showed 23.6 mm^3^. This result confirmed that these mineralized scaffolds were biomimetic and could be used as a substitute to natural bone grafts, a current conventional treatment option. The biomimetic scaffolds produced by mineralization techniques can also overcome some of the drawbacks that natural bone grafts have, such as the immune response associated with rejection in allografts and the secondary surgery needed for autografts. The mineralization approach can be applied to various types of biomaterials including hydrogels, inorganic scaffolds, and fibers, and produces bone scaffolds with improved surface and mechanical properties as well as osteoinductive and osteoconductive properties.

### 4.2. Drug Delivery

Mineralization can also be used for drug delivery applications in vivo, in conjunction with scaffold implantation. This is to help decrease infection rate by providing antibiotics or to promote growth by delivering supporting growth factors. The porosity of composite scaffolds makes these materials suitable delivery vehicles for controlled drug release. For drug loaded mineralized scaffolds, the release profile is tunable, as the release rate of the drug is directly related to the drug’s affinity to the minerals and the degree of mineralization can be controlled. [131]. One example involved the use of CaP coated PLGA microparticle composites for the delivery of Simvastatin [132]. The CaP coating endured the controlled disintegration of the polymer and therefore controlled the drug release. Future studies may be needed for this scaffold to show osteoinductive and osteoconductive properties. In another study, CaP-reinforced, macroporous, chitosan scaffolds were fabricated by a thermally induced phase-separation technique [133]. An antibiotic compound, gentamicin sulphate, was then loaded into the scaffolds. The porous structure of this composite resulted in improved, extended drug release in an in vitro test when compared to the scaffolds without the CaP particles. 

Mineralization is also a feasible method for incorporating water-soluble, anti-bacterial agents into polymer scaffolds [134]. For example, researchers loaded antibiotic ciprofloxacin in a hydroxyethyl methacrylate scaffold, and then incubated the scaffold in SBF for mineralization [135]. The mineralized scaffold was then shown to release the antibiotic, as well as support cell growth and osteogenic differentiation of rat bone marrow mesenchymal cells. Similarly, for alveolar bone regeneration, one group electrospun PLGA/collagen/gelatin nanofibers and mineralized the fibers in 10X SBF [136]. The deposited minerals allowed growth factors (bone morphogenetic protein-2, platelet-derived growth factor, and fibroblast growth factor-2) to be loaded onto the nanofibers. The mineralization, and the released growth factors, promoted the regeneration of bone to fill a critical-sized alveolar defect in a rat model after four weeks. Mineralization on its own not only supports osteogenesis but can also be used to facilitate in-situ drug release. Scaffolds that release small biomolecules such as growth factors, are beneficial for the recruitment, proliferation, and differentiation of cells, which are important for tissue engineering.

## 5. Conclusions and Further Directions

Mineralized scaffolds are promising candidates when it comes to implantable biomaterials for bone repair and regeneration. Future mineralization studies should focus on the mechanisms through which mineralized scaffolds interact with the native environment in order to best improve bone regeneration. Better understanding these mechanisms will likely lead to higher functioning scaffolds. Existing mineralized scaffolds have been shown to improve osteoblast adherence and proliferation, as well as to have osteoinductive properties for the differentiation of pre-osteoblasts and progenitor cells. However, in these reports, researchers did not discuss the possibility that the mineralized scaffolds could also serve as stem cell niches where osteogenic cells could attach and further differentiate in vivo. Furthering scaffolds’ ability to recruit the progenitor cells from the broken end of periosteum would allow the healing process to be accelerated due to intramembranous osteogenesis [137].

The effects of scaffold mineralization on biodegradation, cytotoxicity, and mechanical properties should be carefully evaluated for engineering the bone tissue. Drawbacks can occur when an amorphous mineral has a burst degradation [103] causing a rapid increase in the environment’s calcium ion concentration which can be toxic to the local cells. Other commonly used non-mineralized materials such as metals, ceramics, and polymers will not have this potential limitation. 

The minerals deposited in 3D scaffolds by mineralization processes can disintegrate before scaffold degradation occurs. Further work is necessary to ensure strong adhesion of the minerals to the scaffolds [60,138,139,140,141]. Biodegradation rates influence the rates at which the mechanical properties change over time. An ideal bone scaffold should be able to provide the proper mechanical support for 12 weeks and degrade after there is complete bone regeneration. The material properties of the scaffolds also need to be further studied to optimize the porosity and pore size for cellular applications. The potential immune response to the minerals should also be considered when implementing mineralization [142]. If the mineral particles are not surrounded by bone-forming cells, they could become encapsulated by foreign-body giant cells. In that case, chronic inflammation would significantly hinder regeneration of the bone. The effect of mineralized scaffolds on long bone defects has not yet been investigated extensively. 

Mineralized scaffolds are promising alternatives to non-mineralized biomaterials for bone tissue engineering. The presence of minerals augments the mechanical and surface properties of 3D scaffolds, as well as their osteoinductivity and osteoconductivity, thus improving their regenerative abilities for repairing diseased or damaged bone.

## Figures and Tables

**Table 1:** Representative studies using mineralization approaches for bone regeneration.

Scaffold Substrates	Mineralization Method	Main Experiments	Results	Advantages	Disadvantages	Reference
Type I collagen fiber	Sequential mineralization by calcium and phosphate solutions "	Calculation of steric energies and elastic energy storage	The elastic modulus was improved, and more elastic energy was stored for the mineralized fiber	The method can be applied for various proteins to fabricate true biomimetic material	The mechanical properties are low for these fibers	Landis et al., 2006 [26]
Bacterial cellulose hydrogel	Sequential mineralization by calcium and phosphate solutions	Mechanical testing, XRD, FTIR, and SEM	Calcium-deficient hydroxyapatite was formed	The mineral structure similar to natural bone apatite	The tensile strength was decreased for the mineralized sample and the compressive strength was not tested	Hutchens et al., 2009 [69]
Ti, stainless steel, Si, gold, glass, poly(styrene)poly(methyl methacrylate), polydimethylsiloxane. Si_3_N_4_, filter paper, nylon membrane filter, and polytetrafluoroethylene	Polydopamine was coated on the surfaces of the substrates. The coated ones were then incubated in SBF	SEM, TEM, XRD, high-resolution dispersive Raman microscope and pre-osteoblast culture	Natural inorganic crystalline hydroxyapatite was formed on the surfaces of all substrates	This method enables the hydroxyapatite formation on various synthetic biomaterials with favorable cell viability	For some materials, this method is not the most effective way	Ryu et al., 2010 [60]
Peptide amphiphile (PA) fiber	Combining PA solution with CaCl_2_ solution	Regeneration of a critical-size femoral defect in a rat model	The phosphorylated serine residues promoted mineralization and therefore enhanced bone regeneration	The scaffold can regenerate bone tissue in a defect that will not heal on its own	The mechanical properties are low for these fibers	Mata et al., 2010 [129]
Chitosan/PEGDA hydrogel	Incubation in SBF	SEM, TEM, XRD, FTIR and TGA	Crystalline hydroxyapatite was form on the porous 3D hydrogel network	The bulk mineralization is achieved for the biomimetic scaffold	The in vitro or in vivo study should be performed for the osteoinductivity and osteoconductivity for the scaffold	Zhong and Chu, 2012 [56]
PCL/cellulose acetate nanofiber	Incubation in SBF	Cell viability assay and SEM	The mineralization improved mechanical properties and ensured the excellent cellular compatibility	Osteoblasts showed good attachment on the nanofibers and normal spreading morphology	The mature osteoblasts were directly used in this study. The osteoinductivity remains unknown for this scaffold	Joshi et al., 2015 [58]
PCL/HA coated with calcium phosphate	BMSCs from sheep were cultured and to engineer cell sheet. After the scaffold was implanted in the defect, the BMSCs were the injected to the defect	Regeneration of a critical-size tibia defect in a sheep model	Large amount of regenerated bone tissue and complete bridging of the defect for the BMSC group	This method not only provided the mineralization for scaffolds, but also delivered primary cells	A secondary surgery is needed. Though the cell sheet was injected, the animals were under anesthesia	Berner et al., 2015 [78]
Cellulose grafted soy protein isolate	Incubation in SBF	FTIR, TEM, XRD, TGA, and cytotoxicity tests	Rod-like nanocrystal hydroxyapatite was formed on the scaffold with good biocompatibility	The soy protein isolates successfully modified the cellulose and induced the mineralization	The mechanical testing and bioactivity of the scaffold remain unknown	Salama et al., 2017 [88]
Collagen, chitosan, poly(lactic acid), and poly(lactic-coglycolic acid)	Calcium and phosphate solutions were added to the polymer solution before crosslinking	XRD, SEM, TGA, mechanical testing and biocompatibility testing	The micro-sized porous structure with nanohydroxyapatite crystals were created for the scaffolds with good biocompatibility	The micro-nano structure is beneficial for combining bone morphogenetic protein which will enhance the bone regeneration	The degradation rat of different materials, as long as the formed nHA, may have unexpected impact on bone regeneration	Zou et al., 2019 [40]
Gelatin based hydrogel	Micro-sized eggshells were added to the polymer solution before crosslinking	SEM, mechanical testing, and real-time polymerase chain reaction for osteogenic differentiation makers	The eggshell micro-particles were well embedded in the hydrogel network and induced the osteogenic differentiation of pre-osteoblast	The eggshells improve the mechanical properties of the weak hydrogel substrate and provide natural minerals as a common kitchen waste	The high cell activity induced by the eggshells may result in a faster biodegradation rate of the hydrogel substrate	Wu et al., 2019 [84]

## References

[1] Porter J.R., Ruckh T.T., Popat K.C. (2009). Bone tissue engineering: A review in bone biomimetics and drug delivery strategies. Biotechnol. Prog..

[2] Bolander M.E. (1992). Regulation of Fracture Repair by Growth Factors. Exp. Biol. Med..

[3] Yang F., Yang D., Tu J., Zheng Q., Cai L., Wang L. (2011). Strontium Enhances Osteogenic Differentiation of Mesenchymal Stem Cells and In Vivo Bone Formation by Activating Wnt/Catenin Signaling. Stem Cells.

[4] Logan C.Y., Nusse R. (2004). The Wnt Signaling Pathway in Development and Disease. Annu. Rev. Cell Dev. Biol..

[5] Einhorn T.A. (1998). The Cell and Molecular Biology of Fracture Healing. Clin. Orthop. Relat. Res..

[6] McKibbin B. (1978). The biology of fracture healing in long bones. J. Bone Jt. Surgery..

[7] Giannoudis P.V., Einhorn T.A., Marsh D. (2007). Fracture healing: The diamond concept. Injury.

[8] Lo S.-C., Li K.-C., Chang Y., Hsu M.-N., Sung L.-Y., Vu T.A., Hu Y.-C. (2017). Enhanced critical-size calvarial bone healing by ASCs engineered with Cre/loxP-based hybrid baculovirus. Biomaterials.

[9] Song J., Saiz E., Bertozzi C.R. (2003). A New Approach to Mineralization of Biocompatible Hydrogel Scaffolds: An Efficient Process toward 3-Dimensional Bonelike Composites. J. Am. Chem. Soc..

[10] Camci-Unal G., Laromaine A., Hong E., Derda R., Whitesides G.M. (2016). Biomineralization Guided by Paper Templates. Sci. Rep..

[11] Arora A., Katti D.S. (2016). Understanding the influence of phosphorylation and polysialylation of gelatin on mineralization and osteogenic differentiation. Mater. Sci. Eng. C.

[12] Suvarnapathaki S., Nguyen M.A., Wu X., Nukavarapu S.P., Camci-Unal G. (2019). Synthesis and characterization of photocrosslinkable hydrogels from bovine skin gelatin. RSC Adv..

[13] Rho J.Y., Ashman R.B., Turner C.H. (1993). Young’s modulus of trabecular and cortical bone material: Ultrasonic and microtensile measurements. J. Biomech..

[14] Golebiowska M.A.A., Kim H.S., Camci-Unal G., Nukavarapu S.P. (2019). Integration of Technologies for Bone Tissue Engineering. Reference Module in Biomedical Sciences.

[15] Hancock M.J., Piraino F., Camci-Unal G., Rasponi M., Khademhosseini A. (2011). Anisotropic material synthesis by capillary flow in a fluid stripe. Biomaterials.

[16] Suvarnapathaki S., Wu X., Lantigua D., Nguyen M.A., Camci-Unal G. (2019). Breathing life into engineered tissues using oxygen-releasing biomaterials. NPG Asia Mater..

[17] Chiu Y.-C., Cheng M.-H., Engel H., Kao S.-W., Larson J.C., Gupta S., Brey E.M. (2011). The role of pore size on vascularization and tissue remodeling in PEG hydrogels. Biomaterials.

[18] Yeo A., Wong W.J., Teoh S.-H. (2010). Surface modification of PCL-TCP scaffolds in rabbit calvaria defects: Evaluation of scaffold degradation profile, biomechanical properties and bone healing patterns. J. Biomed. Mater. Res. Part A.

[19] Kim J.-A., Lim J.W., Naren R., Yun H.-S., Park E.K. (2016). Effect of the biodegradation rate controlled by pore structures in magnesium phosphate ceramic scaffolds on bone tissue regeneration in vivo. Acta Biomater..

[20] Taylor B., Indano S., Yankannah Y., Patel P., Perez X.I., Freeman J.W. (2019). Decellularized Cortical Bone Scaffold Promotes Organized Neovascularization In Vivo. Tissue Eng. Part. A.

[21] Wubneh A., Tsekoura E.K., Ayranci C., Uludağ H. (2018). Current state of fabrication technologies and materials for bone tissue engineering. Acta Biomater..

[22] Li J.J., Kaplan D.L., Zreiqat H. (2014). Scaffold-based regeneration of skeletal tissues to meet clinical challenges. J. Mater. Chem. B.

[23] Hjortnaes J., Goettsch C., Hutcheson J.D., Camci-Unal G., Lax L., Scherer K., Body S., Schoen F.J., Kluin J., Khademhosseini A. (2016). Simulation of early calcific aortic valve disease in a 3D platform: A role for myofibroblast differentiation. J. Mol. Cell. Cardiol..

[24] Aidun A., Zamanian A., Ghorbani F. (2018). Novel bioactive porous starch-siloxane matrix for bone regeneration: Physicochemical, mechanical, and in vitro properties. Biotechnol. Appl. Biochem..

[25] Madihally S.V., Matthew H.W. (1999). Porous chitosan scaffolds for tissue engineering. Biomaterials.

[26] Landis W.J., Silver F.H., Freeman J.W. (2006). Collagen as a scaffold for biomimetic mineralization of vertebrate tissues. J. Mater. Chem..

[27] Colosi C., Shin S.R., Manoharan V., Massa S., Costantini M., Barbetta A., Dokmeci M.R., Dentini M., Khademhosseini A. (2015). Microfluidic Bioprinting of Heterogeneous 3D Tissue Constructs Using Low-Viscosity Bioink. Adv. Mater..

[28] Holmes R.E. (1979). Bone Regeneration within a Coralline Hydroxyapatite Implant. Plast. Reconstr. Surg..

[29] Vacanti C.A., Bonassar L.J., Vacanti M.P., Shufflebarger J. (2001). Replacement of an Avulsed Phalanx with Tissue-Engineered Bone. N. Engl. J. Med..

[30] Yuan H., Kurashina K., De Bruijn J.D., Li Y., De Groot K., Zhang X. (1999). A preliminary study on osteoinduction of two kinds of calcium phosphate ceramics. Biomaterials.

[31] Flatley T.J., Lynch K.L., Benson M. (1983). Tissue Response to Implants of Calcium Phosphate Ceramic in the Rabbit Spine. Clin. Orthop. Relat. Res..

[32] Graves G.A., Noyes F.R., Villanueva A.R. (1975). The influence of compositional variations on bone ingrowth of implanted porous calcium aluminate ceramics. J. Biomed. Mater. Res..

[33] Laurencin C.T., El-Amin S.F., Ibim S.E., Willoughby D.A., Attawia M., Allcock H.R., Ambrosio A.A. (1996). A highly porous 3-dimensional polyphosphazene polymer matrix for skeletal tissue regeneration. J. Biomed. Mater. Res..

[34] Hutmacher D.W., Schantz T., Zein I., Ng K.W., Teoh S.H., Tan K.C. (2001). Mechanical properties and cell cultural response of polycaprolactone scaffolds designed and fabricated via fused deposition modeling. J. Biomed. Mater. Res..

[35] Thavornyutikarn B., Chantarapanich N., Sitthiseripratip K., Thouas G.A., Chen Q. (2014). Bone tissue engineering scaffolding: Computer-aided scaffolding techniques. Prog. Biomater..

[36] Zhou G., Chang W., Yu X. (2018). Nanofibrous Nerve Conduits with Pre-seeded Bone Marrow Stromal Cells and Cultured by Bioreactor for Enhancing Peripheral Nerve Regeneration. Regen. Eng. Transl. Med..

[37] Black J. (2005). Biological Performance of Materials.

[38] Gkioni K., Leeuwenburgh S.C., Douglas T.E., Mikos A.G., Jansen J.A. (2010). Mineralization of Hydrogels for Bone Regeneration. Tissue Eng. Part. B Rev..

[39] Bose S., Roy M., Bandyopadhyay A. (2012). Recent advances in bone tissue engineering scaffolds. Trends Biotechnol..

[40] Zou L., Zhang Y., Liu X., Chen J., Zhang Q. (2019). Biomimetic mineralization on natural and synthetic polymers to prepare hybrid scaffolds for bone tissue engineering. Colloids Surf. B Biointerfaces.

[41] Yu S.-H., Cölfen H. (2004). Bio-inspired crystal morphogenesis by hydrophilic polymers. J. Mater. Chem..

[42] Yang F., Wolke J., Jansen J. (2008). Biomimetic calcium phosphate coating on electrospun poly(ε-caprolactone) scaffolds for bone tissue engineering. Chem. Eng. J..

[43] Kim C.W., Kim S.E., Kim Y.W., Lee H.J., Choi H.W., Chang J.H., Choi J., Kim K.J., Shim K.B., Jeong Y.-K. (2009). Fabrication of hybrid composites based on biomineralization of phosphorylated poly(ethylene glycol) hydrogels. J. Mater. Res..

[44] Racquel Z. (1991). Calcium Phosphates in Oral Biology and Medicine. Monogr. Oral Sci..

[45] Ruhé P., Boerman O., Russel F.G.M., Spauwen P., Mikos A., Jansen J. (2005). Controlled release of rhBMP-2 loaded poly(dl-lactic-co-glycolic acid)/calcium phosphate cement composites in vivo. J. Control. Release.

[46] Clarke B. (2008). Normal Bone Anatomy and Physiology. Clin. J. Am. Soc. Nephrol..

[47] Seiffert D. (1996). Detection of vitronectin in mineralized bone matrix. J. Histochem. Cytochem..

[48] Gough J.E., Notingher I., Hench L.L. (2004). Osteoblast attachment and mineralized nodule formation on rough and smooth 45S5 bioactive glass monoliths. J. Biomed. Mater. Res..

[49] Shin K., Acri T., Geary S., Salem A.K. (2017). Biomimetic Mineralization of Biomaterials Using Simulated Body Fluids for Bone Tissue Engineering and Regenerative Medicine. Tissue Eng. Part. A.

[50] Veis A., Dorvee J.R. (2012). Biomineralization Mechanisms: A New Paradigm for Crystal Nucleation in Organic Matrices. Calcif. Tissue Int..

[51] Rowlands A.S., George P.A., Cooper-White J.J. (2008). Directing osteogenic and myogenic differentiation of MSCs: Interplay of stiffness and adhesive ligand presentation. Am. J. Physiol. Physiol..

[52] Wolff J. (1986). The Law of Bone Remodelling.

[53] Limmahakhun S., Oloyede A., Sitthiseripratip K., Xiao Y., Yan C. (2017). Stiffness and strength tailoring of cobalt chromium graded cellular structures for stress-shielding reduction. Mater. Des..

[54] Roseti L., Parisi V., Petretta M., Cavallo C., Desando G., Bartolotti I., Grigolo B. (2017). Scaffolds for Bone Tissue Engineering: State of the art and new perspectives. Mater. Sci. Eng. C.

[55] Zhang X., Wang Y., Van Manh N., Wang H., Zhong X., Lia C. (2016). Synergistic intrafibrillar/extrafibrillar mineralization of collagen scaffolds based on a biomimetic strategy to promote the regeneration of bone defects. Int. J. Nanomed..

[56] Zhong C., Chu C.C. (2012). Biomimetic mineralization of acid polysaccharide-based hydrogels: Towards porous 3-dimensional bone-like biocomposites. J. Mater. Chem..

[57] Kokubo T., Takadama H. (2006). How useful is SBF in predicting in vivo bone bioactivity?. Biomaterials.

[58] Joshi M.K., Tiwari A.P., Pant H.R., Shrestha B.K., Kim H.J., Park C.H., Kim C.S. (2015). In Situ Generation of Cellulose Nanocrystals in Polycaprolactone Nanofibers: Effects on Crystallinity, Mechanical Strength, Biocompatibility, and Biomimetic Mineralization. ACS Appl. Mater. Interfaces.

[59] Zhang H., Fu Q.-W., Sun T.-W., Chen F., Qi C., Wu J., Cai Z.-Y., Qian Q., Zhu Y.-J. (2015). Amorphous calcium phosphate, hydroxyapatite and poly(d, l-lactic acid) composite nanofibers: Electrospinning preparation, mineralization and in vivo bone defect repair. Colloids Surf. B Biointerfaces.

[60] Ryu J., Ku S.H., Lee H., Park C.B. (2010). Mussel-Inspired Polydopamine Coating as a Universal Route to Hydroxyapatite Crystallization. Adv. Funct. Mater..

[61] Tanahashi M., Matsuda T. (1997). Surface functional group dependence on apatite formation on self-assembled monolayers in a simulated body fluid. J. Biomed. Mater. Res..

[62] Yang Q., Song F., Zou X., Liao L. (2017). Preparation and mineralization of a biocompatible double network hydrogel. J. Biomater. Sci. Polym. Ed..

[63] Cowan C.M., Shi Y.-Y., O Aalami O., Chou Y.-F., Mari C., Thomas R., Quarto N., Contag C.H., Wu B., Longaker M.T. (2004). Adipose-derived adult stromal cells heal critical-size mouse calvarial defects. Nat. Biotechnol..

[64] Kokubo T. (1998). Apatite formation on surfaces of ceramics, metals and polymers in body environment. Acta Mater..

[65] Rhee S.-H., Tanaka J. (1999). Effect of citric acid on the nucleation of hydroxyapatite in a simulated body fluid. Biomaterials.

[66] Saiz E., Goldman M., Gomez-Vega J., Tomsia A., Marshall G., Marshall S. (2002). In vitro behavior of silicate glass coatings on Ti6Al4V. Biomaterials.

[67] Du C., Cui F.Z., Zhang W., Feng Q.L., Zhu X.D., de Groot K. (2000). Formation of calcium phosphate/collagen composites through mineralization of collagen matrix. J. Biomed. Mater. Res..

[68] Chen Y., Mak A.F.T., Wang M., Li J. (2006). Composite coating of bonelike apatite particles and collagen fibers on poly L-lactic acid formed through an accelerated biomimetic coprecipitation process. J. Biomed. Mater. Res. Part. B Appl. Biomater..

[69] Hutchens S.A., Benson R.S., Evans B.R., Rawn C.J., O’Neill H. (2009). A resorbable calcium-deficient hydroxyapatite hydrogel composite for osseous regeneration. Cellulose.

[70] Cao Y., Mei M.L., Li Q.-L., Chu C.-H., Chu C.H. (2013). Agarose Hydrogel Biomimetic Mineralization Model for the Regeneration of Enamel Prismlike Tissue. ACS Appl. Mater. Interfaces.

[71] Furuichi K., Oaki Y., Ichimiya H., Komotori J., Imai H. (2006). Preparation of hierarchically organized calcium phosphate–organic polymer composites by calcification of hydrogel. Sci. Technol. Adv. Mater..

[72] Madhumathi K., Shalumon K., Rani V.D., Tamura H., Furuike T., Selvamurugan N., Nair S., Jayakumar R. (2009). Wet chemical synthesis of chitosan hydrogel–hydroxyapatite composite membranes for tissue engineering applications. Int. J. Biol. Macromol..

[73] Douglas T., Wlodarczyk M., Pamula E., A Declercq H., De Mulder E.L.W., Bucko M.M., Balcaen L., Vanhaecke F., Cornelissen R., Dubruel P. (2012). Enzymatic mineralization of gellan gum hydrogel for bone tissue-engineering applications and its enhancement by polydopamine. J. Tissue Eng. Regen. Med..

[74] Tomomatsu O., Tachibana A., Yamauchi K., Tanabe T. (2008). A film of collagen/calcium phosphate composite prepared by enzymatic mineralization in an aqueous phase. J. Ceram. Soc. Jpn..

[75] Flores M.G., Yashiro R., Washio K., Yamato M., Okano T., Ishikawa I. (2008). Periodontal ligament cell sheet promotes periodontal regeneration in athymic rats. J. Clin. Periodontol..

[76] Lee P., Tran K., Zhou G., Bedi A., Shelke N.B., Yu X., Kumbar S.G. (2015). Guided differentiation of bone marrow stromal cells on co-cultured cartilage and bone scaffolds. Soft Matter.

[77] Sikavitsas V.I., Bancroft G.N., Lemoine J.J., Liebschner M.A.K., Dauner M., Mikos A.G. (2005). Flow Perfusion Enhances the Calcified Matrix Deposition of Marrow Stromal Cells in Biodegradable Nonwoven Fiber Mesh Scaffolds. Ann. Biomed. Eng..

[78] Berner A., Henkel J., Woodruff M.A., Steck R., Nerlich M., Schuetz M.A., Hutmacher D.W. (2015). Delayed minimally invasive injection of allogenic bone marrow stromal cell sheets regenerates large bone defects in an ovine preclinical animal model. Stem Cells Transl. Med..

[79] Holzwarth J.M., Ma P.X. (2011). Biomimetic nanofibrous scaffolds for bone tissue engineering. Biomaterials.

[80] Gao T., Zhang N., Wang Z., Wang Y., Liu Y., Ito Y., Zhang P. (2015). Biodegradable Microcarriers of Poly(Lactide-co-Glycolide) and Nano-Hydroxyapatite Decorated with IGF-1 via Polydopamine Coating for Enhancing Cell Proliferation and Osteogenic Differentiation. Macromol. Biosci..

[81] Cui Y., Liu Y., Cui Y., Jing X., Zhang P., Chen X. (2009). The nanocomposite scaffold of poly(lactide-co-glycolide) and hydroxyapatite surface-grafted with l-lactic acid oligomer for bone repair. Acta Biomater..

[82] Stevens M.M. (2008). Biomaterials for bone tissue engineering. Mater. Today.

[83] Petite H., Viateau V., Bensaïd W., Meunier A., De Pollak C., Bourguignon M., Oudina K., Sedel L., Guillemin G. (2000). Tissue-engineered bone regeneration. Nat. Biotechnol..

[84] Wu X., Stroll S.I., Lantigua D., Suvarnapathaki S., Camci-Unal G. (2019). Eggshell particle-reinforced hydrogels for bone tissue engineering: An orthogonal approach. Biomater. Sci..

[85] Menczel J.D., Prime R.B. (2009). Thermal Analysis of Polymers.

[86] Vyazovkin S. (2012). Thermogravimetric Analysis. Characterization of Materials.

[87] Douglas T.E.L., Krawczyk G., Pamuła E., Declercq H.A., Schaubroeck D., Bucko M.M., Balcaen L., Van Der Voort P., Bliznuk V., Vreken N.V.D. (2014). Generation of composites for bone tissue-engineering applications consisting of gellan gum hydrogels mineralized with calcium and magnesium phosphate phases by enzymatic means. J. Tissue Eng. Regen. Med..

[88] Salama A., Shukry N., El-Gendy A., El-Sakhawy M. (2017). Bioactive cellulose grafted soy protein isolate towards biomimetic calcium phosphate mineralization. Ind. Crop. Prod..

[89] Zhou B., He M., Wang P., Fu H., Yu Y., Wang Q., Fan X. (2017). Synthesis of silk fibroin-g-PAA composite using H2O2-HRP and characterization of the in situ biomimetic mineralization behavior. Mater. Sci. Eng. C.

[90] Si J., Cui Z., Wang Q., Liu Q.C., Liu C. (2016). Biomimetic composite scaffolds based on mineralization of hydroxyapatite on electrospun poly(ε-caprolactone)/nanocellulose fibers. Carbohydr. Polym..

[91] Lee S.-D., Hsiue G.-H., Chang P.C.-T., Kao C.-Y. (1996). Plasma-induced grafted polymerization of acrylic acid and subsequent grafting of collagen onto polymer film as biomaterials. Biomaterials.

[92] Chittur K.K. (1998). FTIR/ATR for protein adsorption to biomaterial surfaces. Biomaterials.

[93] Vo T.N., Tatara A.M., Santoro M., Beucken J.J.J.P.V.D., Leeuwenburgh S., Jansen J.A., Mikos A.G. (2016). Acellular mineral deposition within injectable, dual-gelling hydrogels for bone tissue engineering. J. Biomed. Mater. Res. Part A.

[94] Bosch-Reig F., Gimeno-Adelantado J.V., Bosch-Mossi F., Domenech A. (2017). Quantification of minerals from ATR-FTIR spectra with spectral interferences using the MRC method. Spectrochim. Acta Part A Mol. Biomol. Spectrosc..

[95] Shindo D., Oikawa T. (2002). Energy Dispersive X-ray Spectroscopy. Analytical Electron Microscopy for Materials Science.

[96] Liao S., Murugan R., Chan C.K., Ramakrishna S. (2008). Processing nanoengineered scaffolds through electrospinning and mineralization suitable for biomimetic bone tissue engineering. J. Mech. Behav. Biomed. Mater..

[97] Li B., Kan L., Zhang X., Li J., Li R., Gui Q., Qiu D., He F., Ma N., Wang Y. (2017). Biomimetic Bone-like Hydroxyapatite by Mineralization on Supramolecular Porous Fiber Networks. Langmuir.

[98] Stevie F.A., Vartuli C.B., Giannuzzi L.A., Shofner T.L., Brown S.R., Rossie B., Hillion F., Mills R.H., Antonell M., Irwin R.B. (2001). Application of focused ion beam lift-out specimen preparation to TEM, SEM, STEM, AES and SIMS analysis. Surf. Interface Anal..

[99] Cui Y., Chang X., Zhu X., Luo H., Hu Z., Zou X., He Q. (2007). Chemically modified silica gel with p-dimethylaminobenzaldehyde for selective solid-phase extraction and preconcentration of Cr(III), Cu(II), Ni(II), Pb(II) and Zn(II) by ICP-OES. Microchem. J..

[100] Douglas T., Pilarz M., Lopez-Heredia M., Brackman G., Schaubroeck D., Balcaen L., Bliznuk V., Dubruel P., Knabe-Ducheyne C., Vanhaecke F. (2015). Composites of gellan gum hydrogel enzymatically mineralized with calcium-zinc phosphate for bone regeneration with antibacterial activity. J. Tissue Eng. Regen. Med..

[101] Cassetta A. (2014). X-Ray Diffraction (XRD). Encyclopedia of Membranes.

[102] Chen Q., Wong C., Lu W.W., Cheung K.M., Leong J., Luk K. (2004). Strengthening mechanisms of bone bonding to crystalline hydroxyapatite in vivo. Biomaterials.

[103] Chun S.S., Jeong J.H., Kim K.H., Kim S.Y. (2000). Biodegradation Study of Amorphous and Crystalline Calcium Metaphosphate in the SBF and Tris-Buffer Solution. Key Eng. Mater..

[104] Chauhan A. (2014). Powder XRD Technique and its Applications in Science and Technology. J. Anal. Bioanal. Tech..

[105] Nijsure M.P., Pastakia M., Spano J., Fenn M.B., Kishore V. (2017). Bioglass incorporation improves mechanical properties and enhances cell-mediated mineralization on electrochemically aligned collagen threads. J. Biomed. Mater. Res. Part A.

[106] (2012). Nanotechnology Research Methods for Foods and Bioproducts. Nanotechnol. Res. Methods Foods Bioprod..

[107] Burghardt R., Droleskey R. (2006). Transmission Electron Microscopy. Curr. Protoc. Microbiol..

[108] Inkson B. (2016). Scanning electron microscopy (SEM) and transmission electron microscopy (TEM) for materials characterization. Materials Characterization Using Nondestructive Evaluation (NDE) Methods.

[109] Merino S., Novillo C., De Diego G., Conde J.J., Folgado M.A., Ferreira-Aparicio P., Chaparro A.M. (2019). Comparing Different Cross-Section Cutting Methods for SEM Analysis of Membrane-Electrodes Assemblies. ECS Trans..

[110] Ritman E.L. (2004). Micro-Computed Tomography—Current Status and Developments. Annu. Rev. Biomed. Eng..

[111] Bouxsein M.L., Boyd S.K., Christiansen B.A., Guldberg R.E., Jepsen K.J., Muller R. (2010). Guidelines for assessment of bone microstructure in rodents using micro-computed tomography. J. Bone Miner. Res..

[112] Yeo A., Wong W.J., Khoo H.H., Teoh S.H. (2010). Surface modification of PCL-TCP scaffolds improve interfacial mechanical interlock and enhance early bone formation: An in vitro and in vivo characterization. J. Biomed. Mater. Res. Part A.

[113] Lin A.S.P., Barrows T.H., Cartmell S.H., Guldberg R.E. (2003). Microarchitectural and mechanical characterization of oriented porous polymer scaffolds. Biomaterials.

[114] Sijbers J., Postnov A. (2004). Reduction of ring artefacts in high resolution micro-CT reconstructions. Phys. Med. Biol..

[115] Batiste D.L., Kirkley A., Laverty S., Thain L.M., Spouge A.R., Gati J.S., Foster P.J., Holdsworth D.W. (2004). High-resolution MRI and micro-CT in an ex vivo rabbit anterior cruciate ligament transection model of osteoarthritis. Osteoarthr. Cartil..

[116] Mehboob H., Chang S.-H. (2015). Effect of structural stiffness of composite bone plate–scaffold assembly on tibial fracture with large fracture gap. Compos. Struct..

[117] Moreo P., Garcia-Aznar J.M., Doblaré M. (2008). Modeling mechanosensing and its effect on the migration and proliferation of adherent cells. Acta Biomater..

[118] Menard K.P., Menard N. (2017). Dynamic Mechanical Analysis. Encyclopedia of Analytical Chemistry.

[119] Wang J.C. (1984). Young’s modulus of porous materials. J. Mater. Sci..

[120] Ratassepp M., Rao J., Fan Z. (2018). Quantitative imaging of Young’s modulus in plates using guided wave tomography. NDT E Int..

[121] Chartoff R.P., Dillman S.H. (2009). Dynamic Mechanical Analysis (DMA). Thermal Analysis of Polymers.

[122] Storage T.M., Brockman R.A., Tienda K.M. (2013). Analysis of Data Reduction Strategy Used in TA Instruments Q800 DMA Test System. https://apps.dtic.mil/dtic/tr/fulltext/u2/a591605.pdf.

[123] Menard K. (1999). Dynamic Mechanical Analysis.

[124] Wadud S.E.B. (2016). Dynamic Mechanical Analysis and Its Advantages for Deflection Temperature under Load Measurements. TA Instrum.

[125] Rauner N., Meuris M., Zoric M., Tiller J.C. (2017). Enzymatic mineralization generates ultrastiff and tough hydrogels with tunable mechanics. Nat. Cell Biol..

[126] Rath B., Nam J., Knobloch T.J., Lannutti J.J., Agarwal S. (2008). Compressive forces induce osteogenic gene expression in calvarial osteoblasts. J. Biomech..

[127] Sanzherrera J., García-Aznar J.M., Doblare M. (2009). On scaffold designing for bone regeneration: A computational multiscale approach. Acta Biomater..

[128] Pobloth A., Checa S., Razi H., Petersen A., Weaver J.C., Schmidt-Bleek K., Windolf M., Tatai A.Á., Roth C.P., Schaser K.-D. (2018). Mechanobiologically optimized 3D titanium-mesh scaffolds enhance bone regeneration in critical segmental defects in sheep. Sci. Transl. Med..

[129] Mata A., Geng Y., Henrikson K.J., Aparicio C., Stock S.R., Satcher R.L., Stupp S.I. (2010). Bone regeneration mediated by biomimetic mineralization of a nanofiber matrix. Biomaterials.

[130] Nayef L., Mekhail M., Benameur L., Rendon J.S., Hamdy R., Tabrizian M. (2016). A combinatorial approach towards achieving an injectable, self-contained, phosphate-releasing scaffold for promoting biomineralization in critical size bone defects. Acta Biomater..

[131] Oyane A., Yokoyama Y., Uchida M., Ito A. (2006). The formation of an antibacterial agent–apatite composite coating on a polymer surface using a metastable calcium phosphate solution. Biomaterials.

[132] Matsubayashi M., Terukina T., Hattori Y., Otsuka M. (2018). Preparation of Calcium Phosphate Coated Simvastatin-Loaded PLGA Microspheres Dispersed Alginate Hydrogel Beads as a Controlled Drug Delivery Carrier. Key Eng. Mater..

[133] Zhang Y., Zhang M. (2002). Calcium phosphate/chitosan composite scaffolds for controlled *in vitro* antibiotic drug release. J. Biomed. Mater. Res..

[134] Maeno S., Niki Y., Matsumoto H., Morioka H., Yatabe T., Funayama A., Toyama Y., Taguchi T., Tanaka J. (2005). The effect of calcium ion concentration on osteoblast viability, proliferation and differentiation in monolayer and 3D culture. Biomaterials.

[135] Sreeja S., Muraleedharan C., Varma P.H., Sailaja G. (2020). Surface-transformed osteoinductive polyethylene terephthalate scaffold as a dual system for bone tissue regeneration with localized antibiotic delivery. Mater. Sci. Eng. C.

[136] Boda S.K., Almoshari Y., Wang H., Wang X., Reinhardt R.A., Duan B., Wang D., Xie J. (2019). Mineralized nanofiber segments coupled with calcium-binding BMP-2 peptides for alveolar bone regeneration. Acta Biomater..

[137] Gibon E., Lu L., Goodman S.B. (2016). Aging, inflammation, stem cells, and bone healing. Stem Cell Res. Ther..

[138] Singh A.T., Lantigua D., Meka A., Taing S., Pandher M., Camci-Unal G. (2018). Paper-Based Sensors: Emerging Themes and Applications. Sensors.

[139] Lantigua D., Ni Kelly Y., Unal B., Camci-Unal G. (2017). Engineered Paper-Based Cell Culture Platforms. Adv. Heal. Mater..

[140] Wu X., Suvarnapathaki S., Walsh K., Camci-Unal G. (2018). Paper as a scaffold for cell cultures: Teaching an old material new tricks. MRS Commun..

[141] Camci-Unal G., Newsome D., Eustace B.K., Whitesides G.M. (2015). Fibroblasts Enhance Migration of Human Lung Cancer Cells in a Paper-Based Coculture System. Adv. Heal. Mater..

[142] Yokoyama A., Gelinsky M., Kawasaki T., Kohgo T., König U., Pompe W., Watari F. (2005). Biomimetic porous scaffolds with high elasticity made from mineralized collagen—An animal study. J. Biomed. Mater. Res. Part B Appl. Biomater..

